# Limiting factors of peak and submaximal exercise capacity in LVAD patients

**DOI:** 10.1371/journal.pone.0235684

**Published:** 2020-07-09

**Authors:** Libera Fresiello, Steven Jacobs, Philippe Timmermans, Roselien Buys, Miek Hornikx, Kaatje Goetschalckx, Walter Droogne, Bart Meyns

**Affiliations:** 1 Department of Cardiovascular Sciences, Cardiac Surgery, Katholieke Universiteit Leuven, Leuven, Belgium; 2 Institute of Clinical Physiology, National Research Council, Pisa, Italy; 3 Department of Cardiovascular Medicine, University Hospitals Leuven, Leuven, Belgium; 4 Department of Rehabilitation Sciences, Katholieke Universiteit Leuven, Leuven, Belgium; Thomas Jefferson University, UNITED STATES

## Abstract

**Aims:**

Although patients supported with a Continuous-Flow Left Ventricular Assist Device (CF-LVAD) are hemodynamically stable, their exercise capacity is limited. Hence, the aim of this work was to investigate the underlying factors that lead to peak and submaximal exercise intolerance of CF-LVAD supported patients.

**Methods:**

Seven months after CF-LVAD implantation, eighty three patients performed a maximal cardiopulmonary exercise test and a six minute walk test. Peak oxygen uptake and the distance walked were measured and expressed as a percentage of the predicted value (%VO2p and %6MWD, respectively). Preoperative conditions, echocardiography, laboratory results and pharmacological therapy data were collected and a correlation analysis against %VO2p and %6MWD was performed.

**Results:**

CF-LVAD patients showed a relatively higher submaximal exercise capacity (%6MWD = 64±16%) compared to their peak exertion (%VO2p = 51±14%). The variables that correlated with %VO2p were CF-LVAD parameters, chronotropic response, opening of the aortic valve at rest, tricuspid insufficiency, NT-proBNP and the presence of a cardiac implantable electronic device. On the other hand, the variables that correlated with %6MWD were diabetes, creatinine, urea, ventilation efficiency and CF-LVAD pulsatility index. Additionally, both %6MWD and %VO2p were influenced by the CF-LVAD implantation timing, calculated from the occurrence of the cardiac disease.

**Conclusion:**

Overall, both %6MWD and %VO2p depend on the duration of heart failure prior to CF-LVAD implantation. %6MWD is primarily determined by parameters underlying the patient’s general condition, while %VO2p mostly relies on the residual function and chronotropic response of the heart. Moreover, since %VO2p was relatively lower compared to %6MWD, we might infer that CF-LVAD can support submaximal exercise but is not sufficient during peak exertion. Hence concluding that the contribution of the ventricle is crucial in sustaining hemodynamics at peak exercise.

## Introduction

Continuous Flow Left Ventricular Assist Devices (CF-LVADs) are a reliable therapy for the treatment of end-stage heart failure (HF). They have shown to restore patients’ hemodynamics, prevent end organ failure and improve patient’s survival [1]. As more CF-LVAD implanted patients continue to survive for longer durations, in both as a bridge to transplantation and as a destination therapy [2], there is an increased attention on exercise capacity and quality of life, as well as exercise training in order to improve clinical outcomes [3].

CF-LVAD patients can return to light physical activities such as walking, cycling, driving (in some countries) and working [4]. On the other hand, studies have shown that even though exercise capacity improves after implantation, it remains suboptimal: CF-LVAD patients reach a peak oxygen uptake (VO2p) of ~15.2 ml/kg/min [5] corresponding to 40%-50% of predicted exercise capacity [6]. Walking capacity, measured by a six minute walk test (6MWT), is also limited and shows a large variation between 221 m and 406 m after CF-LVAD implantation [7]. The reasons leading to exercise intolerance are complex, multiorgan related, and result from an incomplete reversal of the HF condition [4]. It is important to acknowledge that patients receiving CF-LVAD are affected by end-stage HF, and although their hemodynamic performance improves, they still possess a multitude of impairments such as endothelial dysfunction, respiratory abnormalities [8], deconditioned skeletal muscles, and poor cardiac function [3].

On the other hand, the CF-LVADs have modest sensitivity to preload, thus not allowing to effectively accommodate the increased venous return during exercise. Abrupt augmentation in central filling pressures are observed in CF-LVAD patients from rest to exercise [9], but the amount of benefit that an increase in CF-LVAD speed would bring to exercise capacity is still a matter of debate [10,11].

Overall, CF-LVAD patients seem to exhibit a different relationship between submaximal and maximal exercise capacity when compared to heart failure subjects [12]. This difference might indicate that CF-LVAD therapy is more effective in improving mild physical activities, such as walking, rather than enhancing intense exertion.

At present, no studies have investigated clinical and hemodynamic parameters during peak and submaximal exercise in the same patient population. Thus, the aim of this work was to identify the leading factors of exercise intolerance for %VOp2 (a marker for peak exercise) and %6MWD (a marker for submaximal exercise) in the same CF-LVAD patient population.

## Methods

### Demographic and implantation data

Data was collected retrospectively on patients that received a CF-LVAD between 2009 and 2019 at the University Hospitals of Leuven. The Ethical Committee of the hospital granted approval for this data collection and the need for individual patient consent was waived.

**Fig 1:**
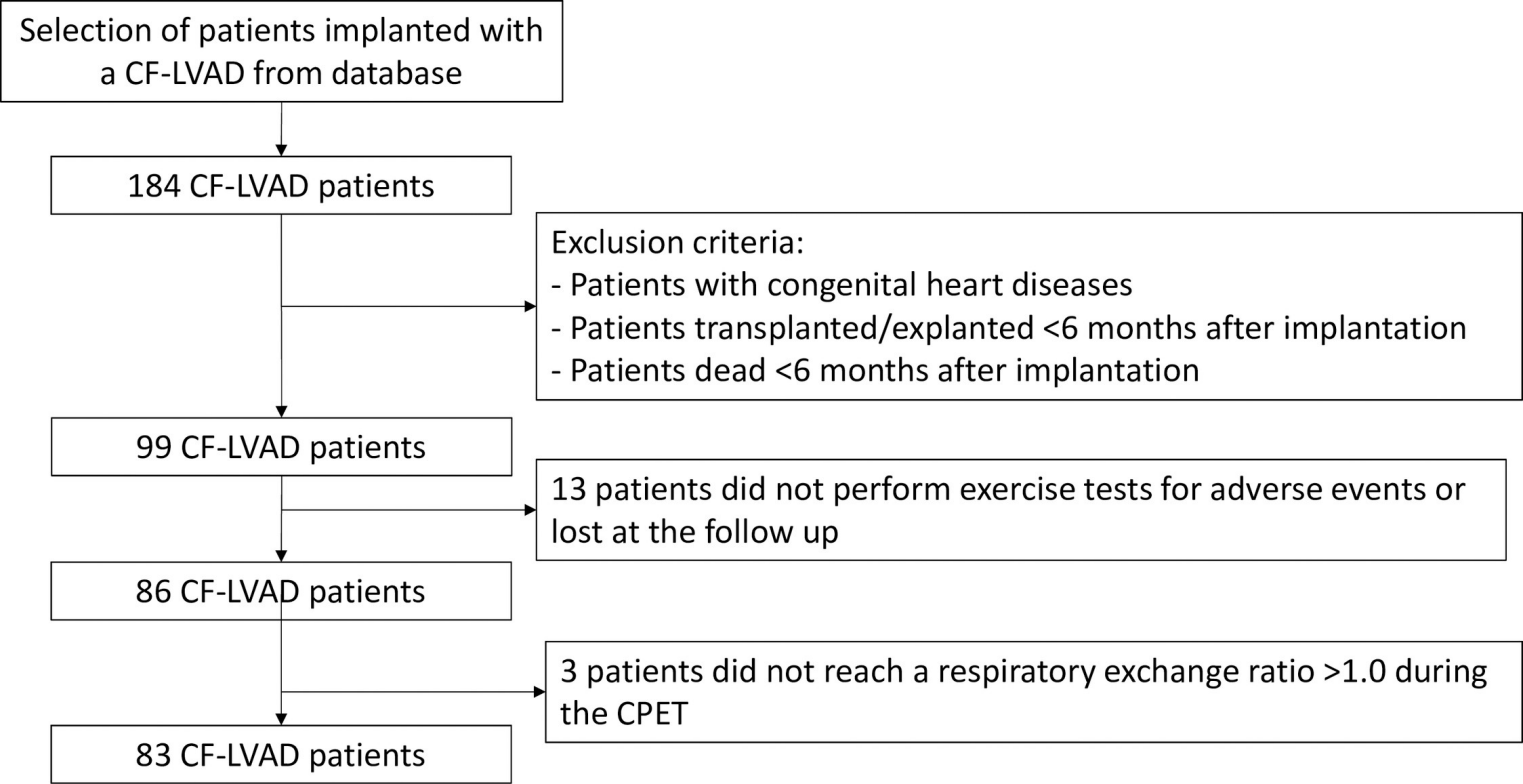
Flow diagram of the selection of CF-LVAD patients’ cohort.

We excluded patients with congenital heart diseases, patients who received a transplant or who were explanted or died before the 6 months follow up after CF-LVAD implantation. Among 99 eligible patients, 13 could not perform the exercise tests due to adverse events or because they were lost at the point of follow up. Additionally, 3 patients exhibited a respiratory exchange ratio of <1.0 during the exercise test, and were also excluded from the analysis as maximal exercise intensity was not reached. A flow diagram of the selection of patients for the study is reported in Fig 1. In total 83 consecutive patients were retained for the analysis.

At LVAD implantation, if there was a preoperative severe aortic insufficiency, the aortic valve was closed using a Park`s stitch. Mitral insufficiency was not treated. Tricuspid repair was only performed in case of structural valve disease. The Interagency Registry for Mechanically Assisted Circulatory Support (INTERMACS) level, the duration of stay in the intensive care unit (ICU) and in the hospital were recorded.

In addition, the date of the first ischemic event (for ischemic patients) or implantation of an electronic device such as pacemaker, implantable cardioverter defibrillator, cardiac resynchronization therapy (for non-ischemic patients) was collected. The number of days between one of these events and the CF-LVAD implantation was retained as an indicator of the duration of heart failure prior CF-LVAD therapy and will be indicated as CF-LVAD timing hereafter.

### Cardiopulmonary exercise test

The cardiopulmonary exercise test (CPET) was performed at 7 months after implantation (220±50 days) on an ergometer bicycle (Ergometrics 800S, Ergometrics, Bitz, Germany) in a laboratory with a stabilized temperature (20–22°C). Patients started cycling at a workload of 10 watts with an increase of 10 watts/minute or at an initial workload of 20 watts with an increase of 20 watts/minute. One or the other protocol was selected according to the results of the last exercise test performed during clinical follow ups. Patients were encouraged to exercise until exhaustion, defined as legs fatigue and/or dyspnea. The CPET exertion level achieved by the patient was assessed by the respiratory exchange ratio. A ratio of ≥1.0 was considered for further analysis, while a value below 1.0 was considered as an indicator of lack of maximal volitional effort [13], and the respective CPET was excluded from the analysis.

A computerized system (Oxygen AlphaR, Jaeger, Mijnhardt, Bunnik, The Netherlands) allowed the continuous recording of respiratory parameters and of oxygen and carbon dioxide concentrations. A 12-lead electrocardiogram was acquired during the entire exercise test (Cardiosoft, CareFusion Corporation, San Diego, California, United States).

Heart rate at rest (HRrest) was obtained by averaging measurements for 60 seconds before the beginning of the test. Peak heart rate (HRp) was defined as the highest value reached during the test. The predicted maximum heart rate was calculated as:

- 220−*age* (in patients not taking β-blockers)
- 119+0.5·HRrest-0.5·age–5 (in patients taking β-blockers) [14].

The heart rate reserve (%HRR) was expressed as a percentage of the ratio between the patient’s heart rate response and the predicted one. Chronotropic incompetence was detected for %HRR<80. The minute ventilation/carbon dioxide production (VE/VCO2) slope was defined as the coefficient of the linear regression analysis performed on the relative variables. It was expressed as percentage of the expected value (%VE/VCO2) calculated according to the patient’s gender and age [15]. When possible, blood pressure was collected manually with a cuff around the upper left arm at rest and at peak exercise. Systolic and diastolic blood pressure were reported at rest (BPSrest, BPDrest), and at peak exercise (BPSp, BPDp).

Peak oxygen consumption was determined as the highest mean value of the last 30 second-interval, and was expressed as percentage of the expected value (%VO2p) according to Wasserman’s equation [16], using the age and weight data collected on the day of the test.

### 6 Minute Walk Test

The 6 Minute Walk Test (6MWT) was also performed 7 months (212±85 days) after CF-LVAD implantation. The covered distance was expressed as percentage (%6MWD) compared to age, weight, height and sex-specific reference standards calculated according to Troosters et al. [17].

### Echocardiographic data

Echocardiographic measurements were performed on GE Healthcare systems at rest on the day of the CPET or within 3 months before or after. The end diastolic and end systolic diameter (LVesDiam, LVedDiam) as well as the ejection fraction (%LVEF) were measured on the left ventricle. The right ventricular functional status was also evaluated, and a global variable was defined (RVfunction) ranging from normal (4) to strongly impaired (0) function. The evaluation took into account the level of dilatation and the contractile properties of the right ventricle.

For the aortic valve, a variable (AVopening) with two categories was created, “yes” and “no”. AVopening was defined as “yes” if the valve was opening even if not completely and not at each cardiac cycle. AVopening was defined as “no” if no significant movements and no forward flow was observed. Mitral, aortic, tricuspid and pulmonary valves insufficiency (MI, AI, TI and PI, respectively) were graded according to ESC/EACVI guidelines on echocardiographic assessment of native valve function [18].

### Laboratory and clinical data

On the day of the CPET, body mass index (BMI) and the age of the patient were collected. Additionally, on the same day, a blood sample was collected via phlebotomy and analyzed for the following: hemoglobin (Hb), serum creatinine, ferritin, NTproBNP, urea and serum albumin.

### Therapy

A dichotomous variable was created to indicate if patients were taking diuretics and β-blockers on the day of the CPET, independent of the type and dosage. Similarly, a dichotomous variable was used to indicate if patients had a cardiac implantable electrophysiological device (CIED, i.e.: pacemaker, implantable cardioverter defibrillator, cardiac resynchronization therapy) regardless of the type. For all these devices, the rate responsive pacing was kept off in presence of the CF-LVAD.

Finally, a patient’s participation in phase II of cardiac rehabilitation after CF-LVAD implantation was also taken into consideration. Specifically, this refers to a supervised training program of 45 sessions starting after hospital discharge.

### CF-LVAD data

Speed, estimated flow, and power consumption CF-LVAD parameters were collected at rest on the day of the CPET. In addition, for the HeartMate II and HeartMate III, the pulsatility index, indicator of the magnitude of pump flow pulse in systole, was collected. Power, speed and pulsatility index data were normalized to the mean and standard deviation value for each pump type.

### Statistical data analysis

#### Entire group analysis

The statistical analysis was executed using the IBM SPSS statistics software (version 23 SPSS Inc., Chicago, IL). Data were checked for normality using the Shapiro-Wilk test. Data was reported as means and standard deviations for normally distributed numerical variables, median (25%, 75%) for not normally distributed numerical variables and as percentage of occurrence for categorical and dichotomous variables.

A Spearman’s rho or a Pearson univariable correlation analysis was performed between %VO2p and each of the considered variables. Point Biserial Correlation analysis was performed between %VO2p and dichotomous variables. Statistical significance was considered for p<0.05. The same analysis was conducted for %6MWD as well.

#### Subgroup analysis

A One-Way ANOVA test was performed to compare %VO2p and %6MWD among patients supported with HVAD, HeartMate II and HeartMate III.

## Results

### Cohort characteristics

The patient cohort included 83 patients, 56 as bridge to transplant and 27 as destination therapy. Within the cohort, majority were male (N = 65, 78%), while the average age of the cohort was 52±15 years and the average body mass index was 25.4±3.9 kg/m^2^ on the day of the CPET. Among the patient cohort, 13 received a HeartWare HVAD device (Medtronic, USA), 50 patients received a HeartMate II device (Abbott, USA) and 20 patients got a HeartMate III device (Abbott, USA). INTERMACS level was 1 in 15 patients, 2 in 23 patients, 3 in 18 patients and 4 in 24 patients. The data collected is reported in Table 1.

**Table 1:** Descriptive analysis of the 83 CF-LVAD patients.

	Variables	N	Mean ± Standard Deviation / Median (25%-75%) /% of occurrence
***Peak and submaximal exercise capacity***			
	%VO2p	83	51±14
	%6MWD	69	64±16
***General***			
	Age, years	83	52±15
	BMI, kg/m^2^	83	25.4±3.9
	Cardiomyopathy	83	49%(ischemic)/51%(non-ischemic)
	Diabetes	83	17%(yes)/83(no)
	Gender	83	78%(males)/22%(females)
	Implantation indication	83	67% (bridge to transplantation)/23% (destination)
	INTERMACS	81	19%(1)/28%(2)/22%(3)/31%(4)
	Days in ICU	83	8 (5–17)
	Days hospitalized	83	28 (20–42)
	Type of CF-LVAD	83	16%(HeartWare)/60%(HeartMate II)/ 24%(HeartMate III)
	CF-LVAD timing, days	79	800 (18–1320)
***Therapy***			
	β-blockers	83	63%
	CIED	83	53%
	Diuretics	83	69%
	Rehabilitation	74	50%
***Blood test***			
	Albumin, g/l	75	44.7±3.5
	Creatinine, mg/dl	83	1.14±0.29
	Hb, g/dl	83	13.2+1.9
	Ferritin, μg/l	45	112 (46–281)
	NTproBNP, ng/l	77	1187 (699–1591)
	Urea, mg/dl	52	40 (32–45)
***Cardiopulmonary exercise test***			
	VO2p, ml/kg/min	83	14.8±4.5
	HRp, bpm	83	131±29
	HRrest, bpm	83	80±15
	%HRR	83	93±48
	BPDrest, mm Hg	73	75±15
	BPSrest, mm Hg	73	106±19
	BPDp, mm Hg	73	82±14
	BPSp, mm Hg	73	125±24
	VE/VCO2	83	38.9±8.6
	%VE/VCO2	83	141±29
	Reasons to stop CPET	66	33% (dyspnea)/35% (legs fatigue)/27%(dyspnea+legs fatigue)/ 5%(other)
***Submaximal exercise test***			
	6MWD, m	69	467±130
***Echocardiographic evaluation***			
	LVesDiam, mm	58	54.8±13.4
	LVedDiam, mm	62	60.6±10.7
	%LVEF	43	15 (10–18)
	RVfunction	69	30%(normal)/17%(mildly impaired)/30%(moderately impaired)/ 22%(poor)
	MI	76	32%(normal)/36%(mild)/14%(moderate)/7%(severe)
	AI	76	39%(normal)/49%(mild)/9%(moderate)/3%(severe)
	TI	75	17%(normal)/52%(mild)/25%(moderate)/5%(severe)
	PI	73	75%(normal)/22%(mild)/3%(moderate)/0%(severe)
	AVopening	73	32%(yes)/68%(no)
***CF-LVAD data (all patients)***			
	CF-LVADflow, l/min	57	4.5 (4.1–4.8)
***CF-LVAD data (CF-LVAD type subgroups)***			
	HeartWare speed, rpm	8	2600 (2440–2600)
	HeartWare power, watts	8	3.3 (3.3–3.7)
	HeartMate II speed, rpm	38	9400 (9200–9600)
	HeartMate II power, watts	38	5.7 (5.1–6.4)
	HeartMate II pulsatility index	38	5.7±1.1
	HeartMate III speed, rpm	20	5400 (5200–5500)
	HeartMate III power, watts	20	3.8 (3.7–4.1)
	HeartMate III pulsatility index	20	4.2±1.5

Data was reported as means ± standard deviations for normally distributed numerical variables, median (25%, 75%) for not normally distributed numerical variables and as percentage of occurrence for categorical variables. AI: aortic valve insufficiency level; AVopening: aortic valve opening; BMI: body mass index; BPSp (BPDp): systolic (diastolic) blood pressure at peak exercise; BPSrest (BPDrest): systolic (diastolic) blood pressure at rest condition; CIED: cardiac implantable electronic device; CF-LVAD timing: number of days between the first ischemic event/ cardiac electronic device implantation and CF-LVAD implantation; CPET: cardiopulmonary exercise test; Hb: hemoglobin; HRp: peak heart rate; HRrest: heart rate at rest condition; %HRR: percentage of heart rate reserve; ICU: intensive care; %LVEF left ventricular ejection fraction; LVesDiam (LVedDiam): left ventricular end systolic (diastolic) diameter; MI: mitral valve insufficiency level; PI: pulmonary valve insufficiency level; RVfunction: right ventricular function; TI: tricuspid valve insufficiency level; VE/VCO2 (%VE/VCO2): (percentage) ventilation over carbon dioxide slope; VO2p (%VO2p): (percentage) peak oxygen uptake; 6MWD (%6MWD): (percentage) distance walked during the 6 minute walk test.

### Peak exercise assessment

Looking at the peak exercise assessment parameters, the mean CPET duration was 472±159 sec, the peak workload was 94±37 watts and VO2p was 14.8±4.5 ml/kg/min corresponding to a %VO2p of 51±14%. All patients reached a respiratory exchange ratio ≥1.0 with a mean of 1.13±0.10. In total, 37 patients were chronotropic incompetent. To verify if muscle atrophy might have caused an early interruption of the CPET before the true HRp was reached, a subgroup analysis was conducted. The %HRR was 103±42% for patients that stopped the CPET due to dyspnea (N = 22), 79±40 for patients that stopped due to legs fatigue (N = 23), and 73±44 for patients that stopped for both reasons (N = 18), p = 0.103. Anaerobic threshold was reached at 40±16% of the expected %VO2p.

#### Submaximal exercise assessment

The distance covered by patients during the 6MWT was 467±130 m corresponding to a %6MWD of 64±16%.

#### CF-LVAD data

The median CF-LVAD flow among all patients was 4.5 (4.1–4.8) l/min. The speed was 2600 (2440–2600) rpm for HeartWare, 9400 (9200–9600) rpm for HeartMate II and 5400 (5200–5500) rpm for HeartMate III. The median pump power was 3.3 (3.3–3.7) watts for HeartWare, 5.7 (5.1–6.4) watts for HeartMate II, and 3.8 (3.7–4.1) watts for HeartMate III. Pulsatility index was 5.7±1.1 for HeartMate II and 4.2±1.5 for HeartMate III.

#### Parameters affecting exercise capacity

The results of the univariable correlation analysis are reported in Table 2. The variables that statistically correlated with %VO2p were: CF-LVAD timing, CIED, NT-pro BNP, %HRR, TI, AVopening, CF-LVAD speed, CF-LVAD power and CF-LVAD pulsatility index. The variables that statistically correlated with %6MWD were: CF-LVAD timing, diabetes, creatinine, urea, %Ve_VCO2, CF-LVAD pulsatility index.

**Table 2:** Correlation analysis between a single clinical parameter and %VO2p and %6MWD.

		%VO2p		%6MWD	
		r	p	r	p
***Peak and submaximal exercise capacity***					
	%VO2p			**0.430**	**0.001**
***General***					
	BMI	-0.11	0.327	-0.14	0.290
	Cardiomyopathy	0.02	0.865	0.04	0.733
	**Diabetes**	-0.21	0.060	**-0.27**	**0.038**
	Implantation indication	-0.32	0.773	0.057	0.662
	INTERMACS	-0.09	0.410	-0.12	0.342
	Days in ICU	0.04	0.709	0.03	0.799
	Days hospitalized	-0.06	0.589	-0.19	0.142
	**CF-LVAD timing**	**-0.32**	**0.005**	**-0.26**	**0.048**
***Therapy***					
	β-blockers	-0.33	0.765	0.07	0.599
	**CIED**	**-0.31**	**0.004**	-0.24	0.059
	Diuretics	-0.86	0.442	0.07	0.580
	Rehabilitation	0.21	0.075	0.23	0.090
***Blood test***					
	Albumin	-0.04	0.705	0.09	0.511
	**Creatinine**	-0.20	0.070	**-0.29**	**0.027**
	Hb	0.14	0.218	0.15	0.244
	Ferritin	-0.11	0.472	-0.03	0.873
	**NT-proBNP**	**-0.22**	**0.054**	-0.12	0.366
	**Urea**	-0.17	0.119	**-0.32**	**0.013**
***Cardiopulmonary exercise data***					
	**%HRR**	**0.34**	**0.002**	0.23	0.068
	BPDrest	0.08	0.494	0.06	0.663
	BPSrest	0.20	0.105	0.17	0.214
	BPDp	0.04	0.772	-0.02	0.910
	BPSp	0.16	0.188	0.14	0.301
	**%VE/VCO2**	-0.19	0.093	**-0.34**	**0.007**
***Echocardiographic data***					
	LVesDiam	-0.20	0.159	0.10	0.571
	LVedDiam	-0.12	0.340	-0.02	0.876
	%LVEF	0.12	0.328	-0.15	0.326
	RVfunction	0.16	0.197	-0.02	0.910
	MI	-0.04	0.716	0.12	0.388
	AI	-0.21	0.068	-0.01	0.954
	**TI**	**-0.35**	**0.003**	-0.05	0.704
	PI	0.05	0.665	0.18	0.187
	**AVopening**	**0.24**	**0.036**	0.00	0.986
***CF-LVAD data***					
	CF-LVADflow	-0.16	0.238	-0.14	0.376
	**CF-LVADspeed norm.**	**-0.28**	**0.024**	-0.16	0.267
	**CF-LVADpower norm.**	**-0.26**	**0.051**	0.08	0.630
	**CF-LVAD pulsatility index norm. (HeartMate II, HeartMate III)**	**0.46**	**0.001**	**0.38**	**0.018**

AI: aortic valve insufficiency level; AVopening: aortic valve opening; BMI: body mass index; BPSp (BPDp): systolic (diastolic) blood pressure at peak exercise; BPSrest (BPDrest): systolic (diastolic) blood pressure at rest condition; CIED: cardiac implantable electronic device; CF-LVAD timing: number of days between the first ischemic event/ cardiac electronic device implantation and CF-LVAD implantation; Hb: hemoglobin; %HRR: percentage of heart rate reserve; ICU: intensive care; %LVEF left ventricular ejection fraction; LVesDiam (LVedDiam): left ventricular end systolic (diastolic) diameter; MI: mitral valve insufficiency level; PI: pulmonary valve insufficiency level; RVfunction: right ventricular function; TI: tricuspid valve insufficiency level; %VE/VCO2: percentage ventilation over carbon dioxide slope; %VO2p: percentage peak oxygen uptake; %6MWD: percentage distance walked during the 6 minute walk test.

#### Peak vs. submaximal exercise

**Fig 2:**
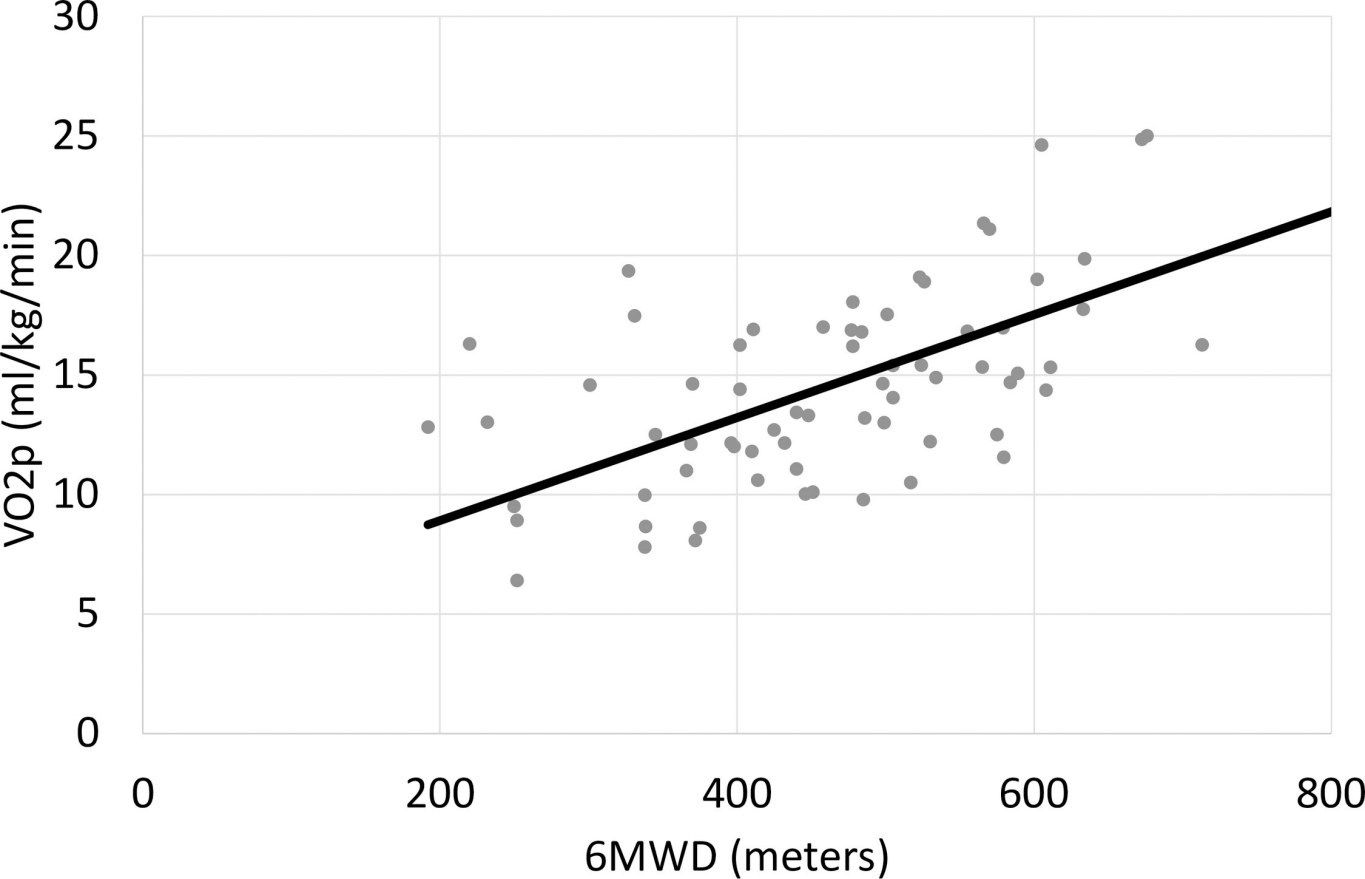
Correlation between 6MWD and VO2p in the CF-LVAD patients’ cohort.

%VO2p and %6MWD were correlated (R = 0.430, p = 0.001) as well as VO2p and 6MWD (R = 0.355, p = 0.006). In Fig 2, the relationship between VO2p and 6MWD for the patient cohort was plotted (VO2p = 0.022·6MWD+4.60).

In addition, we plotted the slope of correlation 6MWD—VO2p in our patients’ cohort and compared it to the slope observed in CF-LVAD patients and HF patients from previous clinical studies [12,19–21].

#### Subgroup analysis

The subgroup analysis did not demonstrate differences in peak exercise capacity among patients supported with the HVAD (%VO2p = 56±14), HeartMate II (%VO2p = 50±12) and HeartMate III (%VO2p = 46±10), p = 0.325. Similarly, no differences were noticed in submaximal exercise capacity among patients with the HVAD (%6MWD = 67±18), HeartMate II (%6MWD = 63±17) and HeartMate III (%6MWD = 67±15), p = 0.196.

## Discussion

In this work the limiting factors of both peak and submaximal exercises were investigated in the same group of CF-LVAD patients. As evidenced by the data, exercise performance is in general influenced by parameters underlying patients’ general condition and by cardiac related parameters. Subgroup analysis did not delineate the differences in exercise capacity among the 3 examined devices (HVAD, HeartMate II and HeartMate III).

Examinations were conducted 7 months after CF-LVAD implantation, as a time point when exercise capacity reaches a plateau and no further improvements are expected [22]. It also corresponds to the time point when CF-LVAD patients conclude their rehabilitation program in our center.

### Limiting factors of submaximal exercise

In the present study, the variables that statistically correlated with %6MWD were: CF-LVAD timing, diabetes, creatinine, urea, %Ve/VCO2, CF-LVAD pulsatility index.

Parameters such as diabetes, creatinine and urea refer to the underlying patients’ general condition and renal function. It was shown that renal function has a transient improvement in the first month after CF-LVAD implantation and then progressively declines to the pre-implant value [23]. The correlation between %6MWT and CF-LVAD timing similarly indicates a worse exercise performance in patients with a more prolonged heart failure prior implantation.

%VE/VCO2 was negatively correlated with %6MWD. A reduced ventilation efficiency (Ve/VCO2>35) was measured in 52 patients (63%). It is unclear whether respiratory function improves after CF-LVAD implantation [24,25], but it is commonly agreed that CF-LVAD patients show an impaired respiratory muscle function and/or ventilation–perfusion mismatch [4].

### Limiting factors of peak exercise

The variables that statistically correlated with %VO2p were: CF-LVAD timing, CIED, NT-proBNP, %HRR, TI, AVopening, CF-LVAD speed, CF-LVAD power, CF-LVAD pulsatility index. Most of these parameters refer to the underlying cardiac function of the patient and are analyzed systematically hereafter.

### Chronotropic response

Our study evidenced a positive correlation between %HRR and %VO2p. Similarly, previous studies have reported the crucial role of chronotropic response in determining exercise performance [26]. In CF-LVAD patients performing peak exercise, heart rate can help the right side in accommodating a higher cardiac output, as well as the left side if the aortic valve starts to open [27]. It is unclear if heart rate can also increase CF-LVAD flow. Muthiah et al. [28] reported no improvements in CF-LVAD flow during a pacing-induced increase in heart rate, but the study was conducted solely during the rest condition.

Given the importance of chronotropic response, it is worth to underline that 37 patients in our study showed chronotropic incompetence, a common condition already reported in CF-LVAD patients [26]. It is worth to mention that the CIED rate responsive pacing was switched off for all the investigated patients, so the device played no role in the chronotropic response measured during the CPET. Even though patients with CIED had a smaller heart rate increase compared to patients without CIED (+46±25 vs. +55±18 bpm, p = 0.049), overall there was no statistical difference found in terms of %HRR (p = 0.237). Among the 37 chronotropic incompetent patients, CIED accounted for 20 of them, while the other 17 had no cardiac electrical device support.

Whether the activation of the rate response could counteract chronotropic incompetence in CF-LVAD patients is an important matter, recently investigated by Alvarez Villela et al. [29]. The clinical study evidenced that the activation of rate response pacing significantly improved 6MWD, while for the CPET less benefits were observed probably due to a lower efficacy in CIED in sensing cycling activity.

Subjects taking β-blocker showed lower heart rates compared to patients not taking β-blocker, both at rest (77±15 bpm vs. 84±14 bpm, p = 0.041) and at peak exercise (137±26 bpm vs. 125±29 bpm, p = 0.064). But the overall increase in heart rate observed during the CPET test was similar (+49±21 bpm vs. +53±24 bpm, p = 0.370). Also, no correlation was found between β-blocker therapy and %VO2p.

To summarize, our data displayed the positive effect of chronotropic response on %VO2p but showed no clear correlation between β-blocker therapy and exercise. In light of these results, further investigations should be conducted to better clarify whether β-blocker dosage can impact inotropic and chronotropic response and in turn exercise, bearing in mind the importance of β-blocker therapy in stimulating myocardial recovery [30,31].

### Left ventricular function

The left ventricular ejection fraction, measured at rest, did not correlate with %VO2p similar to previous studies [32], while Noor et al. [33] found a positive correlation between %LVEF and %VO2p (R = 0.41, p = 0.03). A possible reason for this discrepancy is the difference between the %LVEF in our population (15%) and that of Noor et al. (51±21%). Instead, our analysis revealed that CF-LVAD pulsatility index, a surrogate for left heart function, positively correlated with %VO2p. Moreover, higher values of NT-proBNP were associated with poorer exercise performance. NT-proBNP is an indicator of cardiac congestion [31] and in our cohort it was associated with higher left ventricular end-diastolic and end-systolic volumes (LVesDiam p = 0.048 and LVedDiam p = 0.050) and a higher degree of mitral, aortic and tricuspid valve insufficiency (MI p = 0.012, AI p = 0.001, TI = 0.037, respectively).

An additional cardiac related parameter that correlated with %VO2p was AVopening at rest. AV opening indicates a better contractile function of the left ventricle. We might speculate that these ventricles are also more likely to eject through the aortic valve during exercise, thus contributing to cardiac output increase on top of the CF-LVAD flow [34]. Further, the AVopening at rest correlated with a lower degree of AI (R = -0.286, p = 0.014). It is known that aortic valves opening regularly tend to develop less insufficiency following CF-LVAD [35]. Hence the contribution of AVopening in predicting %VO2p is twofold: it indicates the residual ventricular function and it points to the underlying aortic valve regurgitation, that if present can lead to a blind circulatory loop detrimental for exercise hemodynamics.

### CF-LVAD parameters

%VO2p negatively correlated with CF-LVAD speed and power. Similarly, Rosenbaum et al. showed a correlation between CF-LVAD controller-derived variables and VO2p improvements [24].

In other words, patients in need of a higher CF-LVAD support at rest are the ones who display a poorer performance during the CPET. In our study a higher CF-LVAD support at rest was needed in patients with a poorer left ventricular function (p = 0.050) and larger end-diastolic left ventricular volumes (p = 0.024). As such, it is likely to assume that higher CF-LVAD support at rest indirectly indicate patients with a poorer left ventricle, not capable to support an increase in cardiac output during the CPET.

### Right ventricular function

The negative correlation between TI and %VO2p indicates the role of the right ventricle in influencing exercise performance. Jung et al. reported no association between quantitative evaluation of right ventricular function and VO2p [32]. In Mezzani et al. [36] the right ventricular systolic function was found to be significant in increasing VO2p in response to CF-LVAD speed increase during exercise. To conclude, our results are in favor of a significant role of the right ventricular function on exercise capacity, but previous studies have reported opposing results. As such, more investigations should be conducted to clarify the level of impact the right ventricular function on exercise performance.

### General patient condition

%VO2p negatively correlated with both CIED and the CF-LVAD timing (calculated as the number of days between the first cardiac event reported on patient’s record file and the day of CF-LVAD surgery).

CIED was associated with non-ischemic patients (p<0.001), higher INTERMACS level (p = 0.002) and a longer CF-LVAD timing (p<0.001). Hence, the CIED sorts patients implanted after a long progressive heart failure from patients undergoing an urgent CF-LVAD implantation after cardiogenic shock. The first group is more likely to have a progressive multiorgan deterioration that would ultimately negatively affect exercise performance.

### Peak vs. submaximal exercise

As evidenced by the results, both %6MWD and %VO2p are influenced by the duration of heart failure prior implantation. From these results we can conclude that a long history of heart failure has a general detrimental impact on exercise outcome, both peak and submaximal ones. HF induces multiorgan damages that are not completely reversed by the CF-LVAD therapy, and that are reflected in poorer exercise capacity. With the current generation of devices and decreasing complication rates [37], there is a shift towards earlier CF-LVAD implantation resulting in less kidney failure and more preserved left ventricular function. Ongoing clinical trials using CF-LVADs in higher INTERMACs classes could verify to what extent earlier implantation can be advocated to improve exercise capacity.

%VO2p is also influenced by specific cardiac inotropic and chronotropic parameters, meaning that the residual heart function plays a key role in supporting hemodynamics at peak exercise. This dependence to cardiac related parameters was less present for %6MWD. We can assume that during the submaximal exercise, the CF-LVAD can better sustain the cardiac demand, thus making the patients less dependent on the residual contractile function and inotropic response. It is known that CF-LVAD can “naturally” increase its output during exercise [9, 38] due to the augmentation of preload. This CF-LVAD flow increase can sustain the cardiac output during light physical activity but might be too modest for maximal exertion, so the contribution of the native ventricle remains crucial.

**Fig 3:**
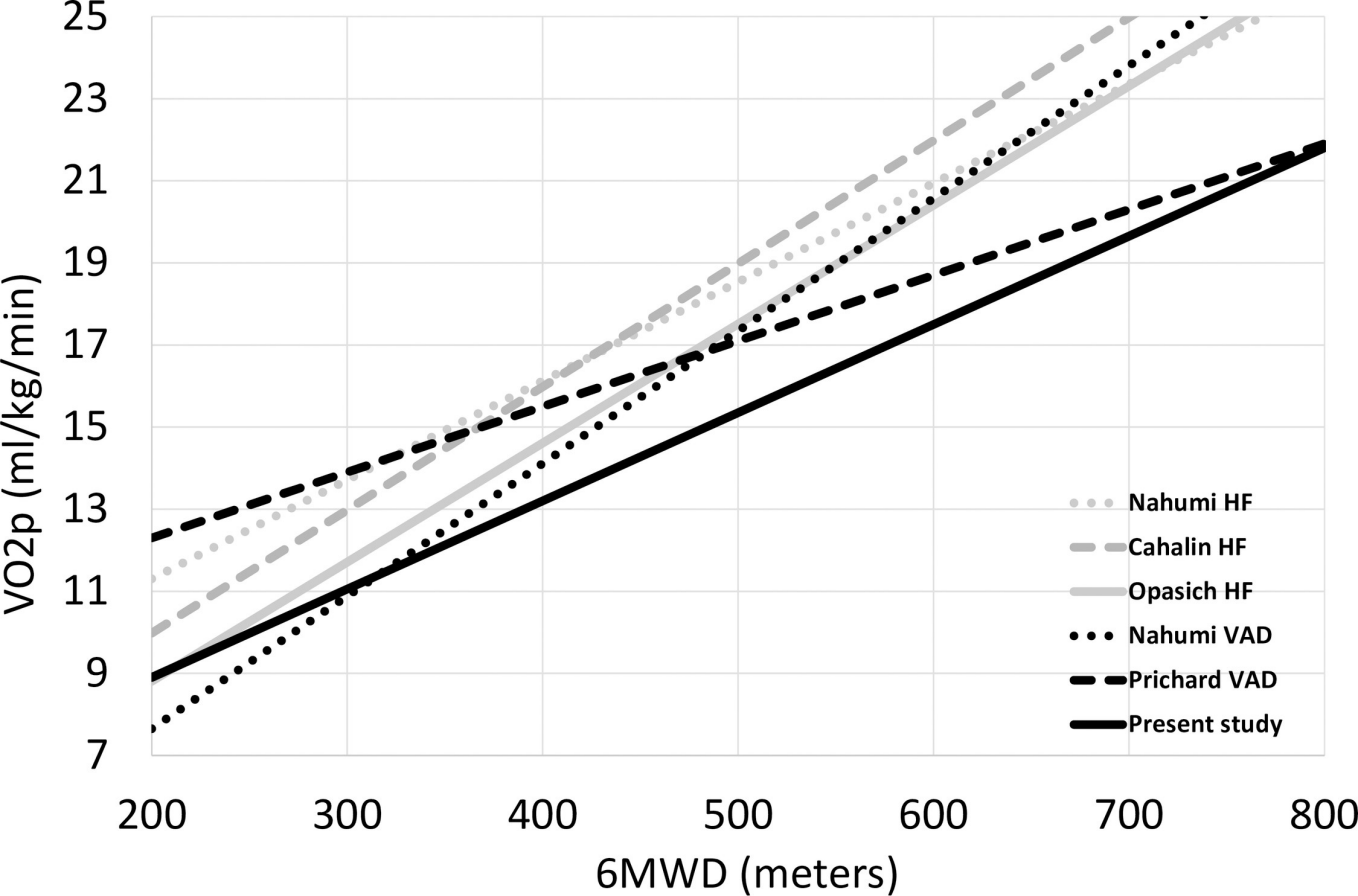
Correlation between 6MWD and VO2p: ●● HF patients from (12), ▬▬ HF patients from (20), ▬▬ HR patients from (19), ● ● CF-LVAD patients from (12), ▬ ▬ CF-LVAD patients from (21), ▬▬ our CF-LVAD patients’ cohort.

This could also explain the relatively better performance of CF-LVAD patients for submaximal exercise than peak exercise. To support this observation, we plotted the slope of correlation 6MWD—VO2p (in Fig 3) and compared it with the slope observed in HF patients from previous clinical studies. We can observe that CF-LVAD patients perform better in 6MWD compared to HF patients for any level of VO2p.

### Study limitations

The monocentric and retrospective designs were the primary limitations of the study. The CPET and the 6MWT were performed at 7 months from CF-LVAD implantation but with a large time variability: 220±50 days for the CPET and 212±85 days for the 6MWT. The CPET and the 6MWT were not conducted on the same day for all patients, with the mean time difference between the two tests being 50±56 days. However, it was shown that peak exercise remains consistent at 6 and 12 months after CF-LVAD implantation [22], so it is likely to assume that the exercise tests were in a time window where the patients reached an almost stable condition. For some patients the echocardiographic investigations were not conducted the same day of the CPET but within 3 months. In addition, the collected database misses some information: the 6MWT was missing in 14 patients, for 10 patients some echocardiographic parameters could not be estimated due to poor image quality, CF-LVAD data were not collected in 17 patients.

Peripheral factors such as the vascular function and the skeletal muscle condition were not considered in the study, and might have contributed to explain the variance of CF-LVAD exercise capacity to a larger extent.

## Conclusions

This study assessed the peak and submaximal exercise (%VO2p and %6MWD) in the same group of CF-LVAD patients. The correlation analysis evidenced that %6MWD and %VO2p are both influenced by the duration of heart failure prior CF-LVAD implantation. %6MWD is mostly influenced by parameters underlying patient’s general condition, %VO2p is mostly influenced by the heart rate reserve and parameters underlying the residual cardiac function. Peak and submaximal exercise are mutually correlated but overall %VO2p is relatively poorer compared to %6MWT. As such, we might infer that CF-LVAD can support submaximal exercise but is not sufficient during peak exertion, so patients also rely on the remaining cardiac function in supporting hemodynamics during maximal exertion.

## List of abbreviations

AI: aortic valve insufficiency level
AVopening: aortic valve opening
BMI: body mass index
BPSp (BPDp): systolic (diastolic) blood pressure at peak exercise
BPSrest (BPDrest): systolic (diastolic) blood pressure at rest condition
CF-LVAD: left ventricular assist device
CF-LVAD timing: number of days between the first ischemic event/ cardiac electronic device implantation and CF-LVAD implantation
CIED: cardiac implantable electrophysiological device
CPET: cardiopulmonary exercise test
Hb: hemoglobin
HF: heart failure
HRp: peak heart rate
%HRR: percentage of heart rate reserve
HRrest: heart rate at rest condition
ICU: intensive care unit
LVEF%: left ventricular ejection fraction
LVesDiam (LVedDiam: left ventricular end systolic (diastolic) diameter
MI: mitral valve insufficiency level
PI: pulmonary valve insufficiency level
RVfunction: right ventricular function
TI: tricuspid valve insufficiency level
VE/VCO2 (%VE/VCO2): (percentage) ventilation over carbon dioxide slope
VErest (VEp): ventilation at rest (peak exercise)
VO2p (%VO2p): (percentage) peak oxygen uptake
6MWD (%6MWD): (percentage) distance walked during the 6 minute walk test
6MWT: 6 minute walk test
